# Enzymatic Regulation of Glycogenolysis in a Subarctic Population of the Wood Frog: Implications for Extreme Freeze Tolerance

**DOI:** 10.1371/journal.pone.0079169

**Published:** 2013-11-13

**Authors:** M. Clara F. do Amaral, Richard E. Lee, Jon P. Costanzo

**Affiliations:** Department of Zoology, Miami University, Oxford, Ohio, United States of America; University of Sao Paulo, Brazil

## Abstract

The wood frog, *Rana sylvatica*, from Interior Alaska survives freezing at –16°C, a temperature 10–13°C below that tolerated by its southern conspecifics. We investigated the hepatic freezing response in this northern phenotype to determine if its profound freeze tolerance is associated with an enhanced glucosic cryoprotectant system. Alaskan frogs had a larger liver glycogen reserve that was mobilized faster during early freezing as compared to conspecifics from a cool-temperate region (southern Ohio, USA). In Alaskan frogs the rapid glucose production in the first hours of freezing was associated with a 7-fold increase in glycogen phosphorylase activity above unfrozen frog levels, and the activity of this enzyme was higher than that of frozen Ohioan frogs. Freezing of Ohioan frogs induced a more modest (4-fold) increase in glycogen phosphorylase activity above unfrozen frog values. Relative to the Ohioan frogs, Alaskan frogs maintained a higher total protein kinase A activity throughout an experimental freezing/thawing time course, and this may have potentiated glycogenolysis during early freezing. We found populational variation in the activity and protein level of protein kinase A which suggested that the Alaskan population had a more efficient form of this enzyme. Alaskan frogs modulated their glycogenolytic response by decreasing the activity of glycogen phosphorylase after cryoprotectant mobilization was well under way, thereby conserving their hepatic glycogen reserve. Ohioan frogs, however, sustained high glycogen phosphorylase activity until early thawing and consumed nearly all their liver glycogen. These unique hepatic responses of Alaskan *R. sylvatica* likely contribute to this phenotype’s exceptional freeze tolerance, which is necessary for their survival in a subarctic climate.

## Introduction

The wood frog, *Rana sylvatica*, is one of several amphibians known to tolerate the freezing of its body fluids as an adaptation to survive winter’s cold [1]. This species is widely distributed in North America, ranging from north of the Arctic circle to as far south as Georgia, USA [2]. Freeze tolerance is likely key to the survival of this amphibian throughout its range because it hibernates in shallow depressions in the soil, under the leaf litter, where it can be exposed to the harsh temperatures of winter. The wood frog can survive the freezing of up to 65–70% of its body water and tolerate temperatures as low as –6°C; however, a recent study [3] reported that wood frogs from Alaska, near the northern limit of their geographic range, can recover from freezing to at least –16°C.

Freeze tolerance in wood frogs is based in part on a glycemic response that is initiated once freezing begins. Glucose, generated from liver glycogen, is quickly exported from the liver and transported to corporal tissues before circulation ceases [4]. Glucose serves as a cryoprotectant by colligatively reducing ice content and cellular dehydration, and by exerting specific effects on membranes and proteins [1]. Glucose improves freeze tolerance at the cellular, tissue, and organismal levels in a concentration dependent manner [5]. The glucose concentration ultimately achieved in frozen tissues is dependent on the size of the hepatic glycogen reserve, rapidity of its catabolism, rate of tissue freezing, and other factors [6].

**Figure 1:**
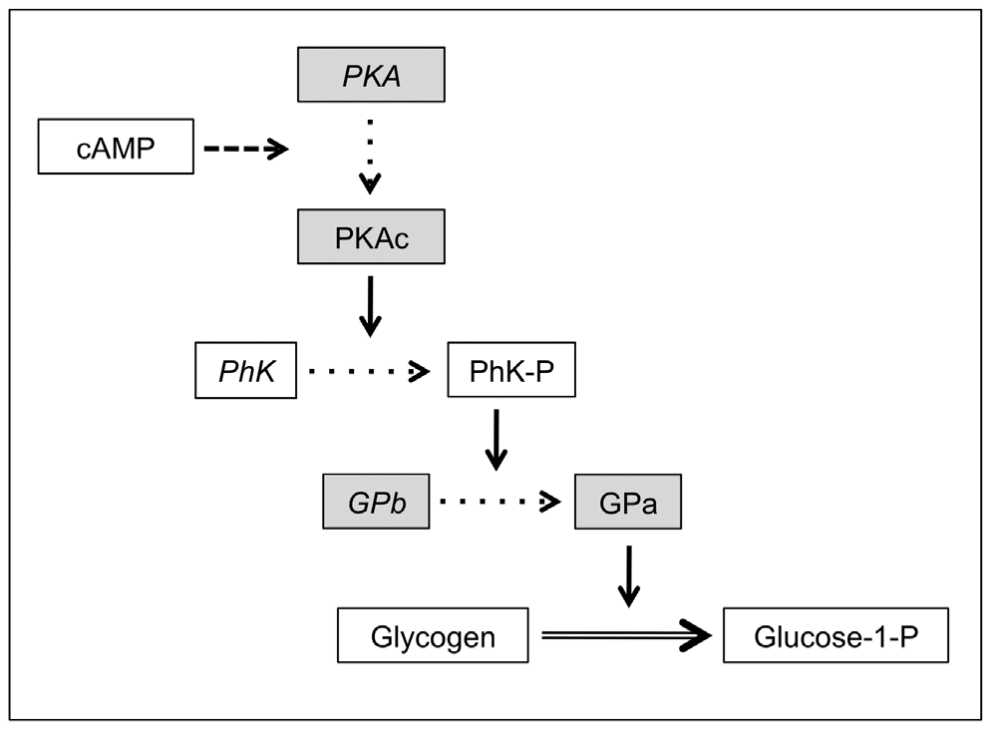
Hepatic glycogenolysis. Simplified representation of the pathway leading to the breakdown of glycogen into glucose in vertebrate liver. Protein kinase A (PKA) and its catalytic subunit (PKAc), glycogen phosphorylase kinase (PhK), glycogen phosphorylase (GPb and GPa) are the main enzymes involved in this process. The pathway involves a phosphorylation cascade, leading to glycogen breakdown. Inactive or dephosphorylated enzymes are italicized. Enzymes assayed in this study are inside grey boxes.

Freezing mobilization of glucose in the wood frog involves β-adrenergic stimulation of hepatocytes, which results in a rapid activation of protein kinase A (PKA, EC 2.7.11.11) in the first minutes of freezing [7]. Binding of four cAMP molecules to the two regulatory subunits of PKA activates the enzyme by releasing the two catalytic subunits (PKAc) from the inactive tetrameric holoenzyme [8]. PKA phosphorylates glycogen phosphorylase kinase (PhK, EC 2.7.11.19), which in turn phosphorylates glycogen phosphorylase (GP, EC 2.4.1.1) [4]. Phosphorylation of GP converts the inactive form of the enzyme, GPb, to the active form, GPa, triggering glycogenolysis and consequent production of the cryoprotectant glucose (Figure 1) [9]. Efficiency of this enzymatic pathway is essential to allow the distribution of glucose throughout the blood and tissues early in the freezing process, as cryoprotectant distribution is curtailed as freezing progresses and ice accumulates [10].

The capacity for freeze tolerance depends in large part on the cryoprotectant levels that can be reached in the corporal tissues [5], [11], [12]. No single study has compared the glycemic freezing response of wood frogs from different populations; however, other freeze-tolerant ectotherms that rely on a glucosic cryoprotectant system show larger glycogen reserves in northern populations and these populations can mobilize more cryoprotectant during freezing as compared to southern populations [12]–[14]. To rapidly achieve high glucose concentrations in the tissues would be advantageous for northern populations, given that they would likely experience much lower winter temperatures. Furthermore, all else being equal, northern frogs would tend to freeze faster as they are smaller than their southern counterparts, resulting in less time for cryoprotectant to reach tissues [2], [10], [15].

The purpose of this study was to examine the glucosic cryoprotectant system of a wood frog population from near the northern limit of this species’ range. Frogs from Interior Alaska tolerate much lower temperatures than frogs from more temperate locations, such as Ohio and Pennsylvania [3]. We hypothesize that the higher freeze tolerance of this northern population is in part the result of an enhanced cryoprotectant system. We tested this hypothesis by using a comparative approach, analyzing the hepatic freezing response in Alaskan and Ohioan wood frogs during freezing and thawing by measuring activity and protein levels of GP and PKA in liver. Additionally, we examined the quantity of glycogen in liver, and its turnover during freezing, to ascertain whether differential responses of key enzymes and carbohydrates could be responsible for the enhanced freeze tolerance of the Alaskan population.

## Materials and Methods

### Ethics Statement

Wood frogs were collected near Fairbanks, Alaska, USA (64.8°N, 147.7°W), on public land, during early August, 2011, under a permit issued by the Alaskan Department of Fish and Game. Additional *R. sylvatica* were collected in late winter (February, 2011) in Adams County, south-central Ohio, USA (38.8°N, 83.3°W), on private land with the permission of the landowner, under a permit issued by the Ohio Division of Wildlife. Frogs were collected by hand or using a dip net. Experimental frogs were euthanized by double-pithing before being dissected. Rearing and experimental protocols were approved by the Institutional Animal Care and Use Committee (IACUC) of Miami University (research protocol number 812).

### Experimental Animals and Acclimatization

Alaskan frogs were held in a programmable environmental chamber (Percival, model I-35X; Boone, IA, USA) and exposed over 5 weeks to dynamic, diel cycles of temperature and ambient light, which, based on long-term records of weather (obtained from the National Oceanic and Atmospheric Administration’s National Climatic Data Center, NOAA NCDC), were seasonal and appropriate to their origin. Initially, temperature varied daily from 17 to 8°C and the photophase was 16.5 h, but by the end of acclimatization, in mid September, temperature varied daily from 13 to 3°C and the photophase was 13.3 h. Throughout, frogs were fed three times weekly with crickets that were dusted with a vitamin supplement (ReptoCal, Tetrafauna, Blacksburg, VA, USA). Following acclimatization, frogs were kept at 4°C, in darkness, in simulated hibernation until used in mid November.

Ohioan frogs were kept, unfed, on damp moss within darkened plastic boxes (4°C) for 3 weeks after collection from the field. Thereafter they were kept outside in a 48-m^2^ pen at the Ecology Research Center (39.5°N, 84.7°W), Miami University, until autumn. Frogs had access to a pool of water and were fed vitamin-fortified crickets three times weekly, and this diet was supplemented by a host of arthropods that was attracted to a “black light” hung in the pen. Feeding was suspended in late October, and in November, the frogs, on the verge of dormancy, were recaptured and kept at 4°C, in darkness, in simulated hibernation until used in January.

We aimed to sample only adult males to eliminate potential gender- and age-based differences in physiology. This objective was largely achieved in the Ohioan frog samples. However, as secondary sex characteristics were not evident in August, Alaskan frogs collected and used in this study comprised about 37% females, which were randomly distributed amongst treatment treatments.

### Experimental Freezing and Thawing

Frogs used in this experiment were frozen and thawed following an established protocol that facilitates cryoprotective responses, promotes survival, and mimics natural freezing and thawing episodes [16]. Prior to freezing, bladder fluid was removed and the standard body mass of each frog was measured. Each frog was placed in a 50-ml polypropylene tube with an insulated thermocouple probe positioned against its abdomen. Throughout the experiment, body temperature (*T*_b_) was recorded at 30-s intervals on a multichannel data logger (Omega, model RD3752; Stamford, CT, USA). Tubes containing these frogs were submerged to the cap in a refrigerated bath (Neslab, model RTE 140; Portsmouth, NH, USA) containing ethanol. After each frog had supercooled slightly (*T*_b_∼–1°C), freezing was initiated by inoculating the skin with small ice crystals created by applying aerosol coolant to the exterior of the tube. Initiation of freezing was confirmed by the occurrence of a freezing exotherm that resulted from the change in the physical state of body water. The temperature of the bath decreased 0.05°C h^–1^ to the target temperature, –2.5°C, which was reached after 30 h; some frogs were kept frozen for an additional 18 h, remaining frozen for a total of 48 h. Groups of frogs (*N* = 5–6) were removed from the bath and sampled at 2, 6, 30, and 48 h after freezing commenced. Other frogs were frozen for 48 h and sampled after thawing at 4°C for either 6 h (*N* = 5–6), or 120 h (5 d, *N* = 4–6). A reference group of unfrozen frogs (*N* = 7–8) was sampled directly from their holding cups (or plastic boxes) in the 4°C chamber.

### Tissue Harvesting

Frogs were weighed to 0.1 g, double-pithed, and dissected at 4°C. The liver was excised rapidly (i.e., within 2 min) and a small sample of tissue was blotted to remove excess moisture, weighed to 0.01 mg, and placed in a 65°C oven for 3–5 d, to determine tissue dry mass. The remaining tissue, destined for enzymatic, protein, and metabolite analyses, was immediately frozen in liquid N_2_. Initial water concentration in the tissue, expressed as mg water g^–1^ dry matter, was determined by dividing the mass lost upon drying the sample by its dry mass. Liver samples were stored at –80°C before enzyme assays and metabolite analyses were carried out.

### Metabolite Analyses

Deproteinized extracts of liver samples were prepared by homogenization in cold 7% (w/v) perchloric acid. Liver samples were assayed for glycogen using an enzymatic procedure [17]. Briefly, a separate portion of the whole-tissue homogenate (100 µl) was neutralized with KOH and incubated with amyloglucosidase (1 mg ml^−1^) at 40°C for 2 h in a 0.2 M sodium acetate buffer, pH 4.8. After incubation, the reaction was stopped by adding cold 7% (w/v) perchloric acid and the free glucose in the sample was determined using a colorimetric assay kit (Pointe Scientific, Canton, MI, USA); glycogen concentration was expressed as µmol glucosyl units g^−1^ dry liver tissue after subtraction of initial free glucose, assayed as above, in the initial homogenate. We also expressed glycogen levels on a µmol liver^−1^ basis, calculated by multiplying the liver glycogen concentration by the dry mass of the entire liver, thus obtaining an estimate of the organ’s total glycogen content. We estimated the cumulative glucose output from the liver during freezing by subtracting the mean liver glycogen remaining at each sample time from the mean liver glycogen content of unfrozen frogs.

### Enzyme Activity Assays

PKA activity was measured in liver extracts using a non-radioactive assay kit (PepTag®, Promega, Madison, WI, USA) that determines phosphotransferase activity with fluorescent kemptide (PepTag® A1 peptide) as the substrate. Frozen liver tissue was homogenized (1∶10) on ice in cold Tris-HCl buffer (pH 7.4) and centrifuged at 4°C (14,000 g, 5 min), per the manufacturer’s instructions. To determine activity of the free catalytic subunit of PKA (PKAc activity), samples were assayed without cAMP; additionally, samples were assayed with 1 µM cAMP to release the catalytic subunits bound to the regulatory subunits, thereby providing a measure of total PKA activity [7]. Additionally, samples were run with and without PKI, a synthetic PKAc inhibitor, to determine any non-specific activity, which was then subtracted from measures of PKAc and total PKA activity [18]. Preliminary experiments showed that a final concentration of 10 µM of PKI resulted in maximal inhibition in our samples. Non-specific activity averaged 20.4±0.8% of total PKA activity.

PKA activity was assayed at 22°C for 30 min, after which the reaction was stopped by boiling the samples at 95°C for 10 min. Phosphorylated and non-phosphorylated PepTag® A1 peptides were separated by 0.8% agarose gel electrophoresis. The fluorescence of the phosphorylated peptides was recorded with an Alpha Inotech Imager (ProteinSimple, Santa Clara, CA) and the bands were analyzed by densitometry with the use of a standard curve of fluorescent kemptide present in each gel. Each liver extract was run in duplicate and the average of the activity values was taken to represent the individual. The percentage of PKA present as the free catalytic subunit was calculated by dividing the activity of PKAc by the total PKA activity and multiplying the ratio by 100. Liver extracts from the unfrozen group were also assayed for total PKA activity at 0°C. The reaction was set up as described above, except that the incubation was done for 30 min on ice. We used the activities measured at 22°C and at 0°C to compute the temperature coefficient (Q_10_) for total PKA activity. Enzyme activity was expressed as U mg^−1^ of total soluble protein, where one Unit was defined as the number of nanomoles of phosphate transferred to the substrate per minute.

GP activity was measured at 22°C using a coupled-enzyme assay under conditions detailed by Swanson et al. [19]. Liver tissue was homogenized (1∶10) on ice in cold imidazole-HCl buffer (pH 7.5) containing inhibitors of protein kinases and phosphatases that preserve the enzyme’s phosphorylation state. GPa activity was determined spectrophotometrically from the change in absorbance caused by conversion of NADP to NADPH, which is coupled with the GP-catalyzed breakdown of glycogen. Total activity of GP (GPa+ GPb) was determined in a separate trial in which the reaction medium included AMP at a final concentration of 1.6 mmol l^−1^ [19]. Each liver extract was run in duplicate and the average of the activity values was taken to represent the individual. The percentage of enzyme present as GPa was calculated by dividing the activity of GPa by the total GP activity and multiplying the ratio by 100. Liver extracts from the unfrozen group were also assayed for total GP activity at 0°C. The reaction was set up as described above, except that the absorbance was measured using a water-jacketed cuvette holder through which chilled ethanol was circulated. The temperature of the assay mixture determined at the beginning and end of the assay was 0°C. We used the activities measured at 22°C and at 0°C to compute the Q_10_ for total GP activity. Enzyme activity was expressed as U mg^−1^ of total soluble protein, where one Unit formed 1.0 µmol of a-D-glucose 1-phosphate from glycogen and orthophosphate per minute.

For each liver extract, the supernatant was assayed for protein concentration using the Bio-Rad protein assay (Bio-Rad, Hercules, CA) with bovine serum albumin (BSA) as the standard.

### Immunoblotting

Liver tissue collected from unfrozen, 48 h-frozen, and 120 h-thawed frogs was homogenized whilst frozen in RIPA buffer (1∶10), on ice, containing a protease-inhibitor cocktail (cat. # P2714, Sigma-Aldrich Chemical Company, Saint Louis, MO, USA). The homogenate was centrifuged (3,000 g for GP; 14,000 g for PKAc) for 5 min at 4°C. Following centrifugation, the supernatant was assayed for protein concentration using the Bio-Rad protein assay (Bio-Rad), with bovine serum albumin (BSA) as the standard, aliquoted, and frozen at −80°C.

Total soluble protein (30 µg for GP; 15 µg for PKAc) was mixed with Laemmli sample buffer (containing 5% β-mercaptoethanol) and heated at 95°C for 5 min. SDS-PAGE of the protein samples was performed using a 4–15% Tris-HCl gradient gel (Bio-Rad). To allow comparisons among different gels, we loaded into all gels a standard relative to which all protein bands were normalized.

Following electrophoresis, proteins were transferred to a nitrocellulose membrane (GE Healthcare, Waukesha, WI, USA). After the transfer, each membrane was stained with 0.2% (w/v) Ponceau S (Sigma-Aldrich) containing 5% (v/v) acetic acid to verify uniformity of protein transfer; these membranes were digitized for densitometry analysis of the total protein loaded. After the membranes were digitally scanned, they were destained using 0.1 M NaOH, rinsed for 3 min with ultrapure water, and blocked overnight in 10% non-fat milk in TBS-T buffer (10 mmol l^−1^ Tris, 500 mmol l^−1^ NaCl, and 0.1% Tween-20; pH 7.5). Goat primary antibody for GP (Santa Cruz Biotechnologies, Santa Cruz, CA, USA) was used to detect total GP in liver samples; rabbit primary antibody for PKAc (Abcam, Cambridge, MA, USA) was used to detect PKAc in liver samples. Nitrocellulose membranes were incubated in anti-GP antibody solution (1∶2000 dilution) or anti-PKAc antibody solution (1∶3000) overnight, at 4°C, with constant oscillation. Secondary antibodies were horseradish peroxidase-linked donkey anti-goat for GP (Santa Cruz Biotechnologies) and goat anti-rabbit for PKAc (Sigma-Aldrich). All primary and secondary antibodies were diluted in a 5% non-fat milk TBS-T solution. Following three 15-min washes in TBS-T, membranes were incubated in secondary antibody (1∶3000) for 2 h at room temperature with constant oscillation. Membranes were thrice washed in TBS-T for 15 min, incubated for 2 min in ECL (enhanced chemiluminescence) detection reagents (GE Healthcare), and exposed to autoradiography film.

PKAc and GP antibodies were exposed to their respective antigenic peptides in a competition assay to determine the specificity of the bands visible in the blot (results not shown). We analyzed only the bands that were specific to each antibody.

Bands from digitally scanned radiography films were semi-quantified using AlphaView spot densitometry (ProteinSimple). We had intended to use β-tubulin as a loading control but found that the β-tubulin concentration in livers of unfrozen Alaskan frogs was about double that of the unfrozen Ohioan frogs (*t* = 2.683, *P* = 0.028, data not shown); thus, we instead elected to use total protein as a loading control. Following Aldridge et al. [20], a densitometric measurement of total protein was performed on the scanned membranes by selecting a thin strip in the center of each lane that encompassed all proteins in the lane. For densitometry of the probed proteins, target bands were selected using a uniform sampling area that encompassed the band of interest. Background optical density was determined individually for each band. Densitometry values determined for individual protein bands were standardized to total protein densitometry values. These ratios were divided by the standard present in all gels, thus allowing comparisons among them. Each membrane contained samples from unfrozen, 48 h-frozen, and 120 h-thawed groups representing both populations, and was run in duplicate.

### Statistical Analysis

Means ± standard error of the mean (SEM) are the descriptive summaries used for the variables measured. Mean values for samples of unfrozen, frozen, and thawed frogs were compared within each population using a one-way ANOVA; post-hoc Dunnett’s test distinguished the mean for each group of frozen or thawed frogs from that for unfrozen frogs. A two-way ANOVA was used to examine the mean response of the variables as a function of two factors, population (Alaskan and Ohioan) and sample treatment (unfrozen, frozen for 2 h, 6 h, 30 h, or 48 h, and thawed for 6 h or 120 h), or the interaction of these two factors. If a significant interaction between the factors was found, Bonferroni post-hoc test was used to distinguish between the two population means for each sample treatment. Mean values for the enzyme activity at 0°C and for the thermal coefficient of enzyme activity were compared between Alaskan and Ohioan frogs using the Student’s *t*-test. As necessary, data were transformed to the natural logarithm to fulfill the parametric tests’ assumptions. Analyses were performed using SYSTAT (Cranes Software International Limited, Chicago, IL); significance was accepted at *P*<0.05.

## Results

Exotherms were observed in the temperature recordings of all frogs subjected to experimental freezing, confirming that frogs from both populations were successfully frozen. Upon dissection, Alaskan frogs contained relatively little ice, even after 48 h of freezing, which was present beneath the skin ventral to the submaxillary and rectus abdominis muscles. Frozen Ohioan frogs had large, subcutaneous ice crystals present both dorsally and ventrally along the body, in the coelom, and also between muscle fibers of the hind limbs. Overall, Alaskan frogs were much more pliable as compared to frozen Ohioan frogs, which were more rigid. Survival was assessed in the 120 h-thawed groups before they were dissected for tissue sampling. All frogs survived experimental freezing and thawing except for one Alaskan frog, which was eliminated from the study.

### Liver Metabolites

**Figure 2:**
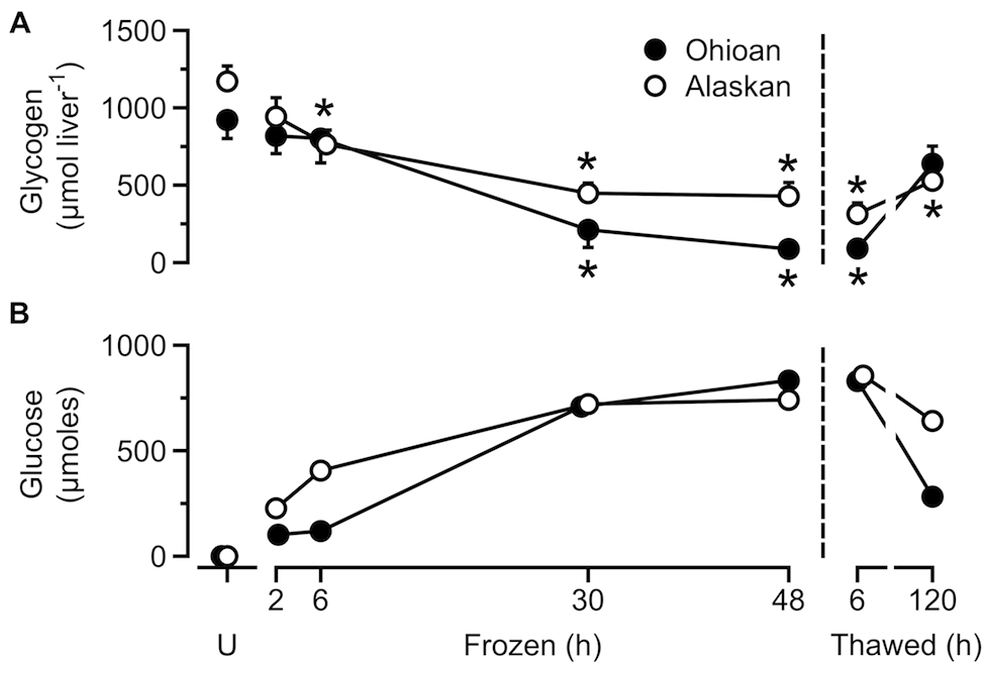
Hepatic glycemic response during freezing and thawing. Liver glycogen content (A) and glucose output (B) during freezing and thawing in Alaskan and Ohioan *R. sylvatica* as compared to that in unfrozen frogs (U) (mean ± SEM; *N* = 4–8). Glucose output (B) was calculated by subtracting the mean liver glycogen content (µmol liver ^−1^) remaining at each sample from the mean liver glycogen content of unfrozen frogs. Asterisk indicates that the value differs from the mean for the corresponding sample of unfrozen frogs (Dunnett’s, *P*<0.05). Some overlapping values were slightly offset along the abscissa for clarity.

Liver glycogen levels varied throughout the experiment in both frog populations (Alaskan, *F*_6,35_ = 13.06, *P*<0.0005; Ohioan, *F*_6,29_ = 11.52, *P*<0.0005; Figure 2a). Liver glycogen decreased (*P*<0.01) noticeably in Alaskan frogs as early as 6 h of freezing, but glycogenolysis slowed as freezing progressed, and these frogs consumed only 63% of their initial glycogen store by 48 h of freezing. On the other hand, Ohioan frogs first showed a significant (*P*<0.0005) decrease in liver glycogen by 30 h of freezing, and by 48 h had consumed 90% of their initial glycogen store. Overall, Alaskan frogs had a higher (*F*_1,64_ = 7.24, *P* = 0.009) liver glycogen content than Ohioan frogs, indicating that their potential to mobilize glucose during freezing could be greater if GP activity is also comparatively higher during early freezing. The pattern of glucose output, deduced from the time course of glycogen depletion, showed that Alaskan frogs mobilized glucose more rapidly in the early stages of freezing, although by 48 h both populations had produced identical amounts of glucose (Figure 2b).

### Liver PKA

**Figure 3:**
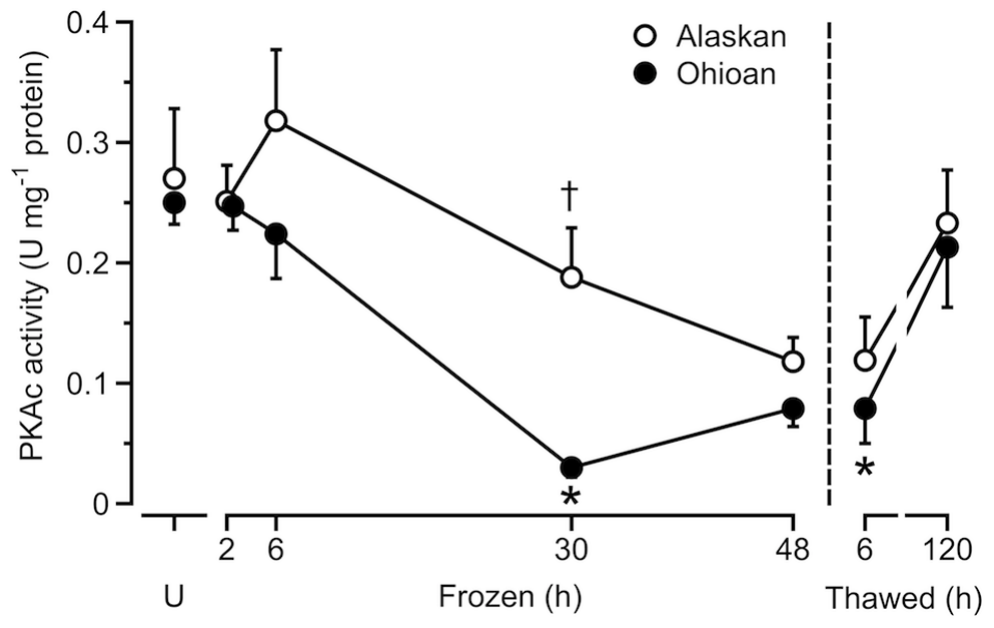
Activity of liver PKAc during freezing and thawing. Activity of hepatic PKAc (mean ± SEM; *N* = 4–8) in frozen and thawed Alaskan and Ohioan *R. sylvatica* as compared to that in unfrozen frogs (U). Asterisk indicates that the value differs from the mean for the corresponding sample of unfrozen frogs (Dunnett’s, *P*<0.05); dagger indicates that the value differs between populations (Bonferroni, *P*<0.05). Some overlapping values were slightly offset along the abscissa for clarity.

Activity of PKAc in the Alaskan frogs varied significantly (*F*_6,35_ = 2.72, *P* = 0.028), although no sample treatment had an activity level that differed from that of the unfrozen frogs (Figure 3). Activity of PKAc also varied (*F*_6,29_ = 13.05, *P*<0.0005) with freezing/thawing in the Ohioan frogs, decreasing to 12% of the values of unfrozen frogs by 30 h; however, activity levels were restored to basal values by 120 h of thawing (Figure 3). Results of the ANOVA showed that Alaskan frogs had a higher (*F*_1,64_ = 6.3, *P* = 0.015) activity of PKAc.

Total PKA activity did not vary throughout the experiment in either population (Alaskan, *F*_6,35_ = 0.70, *P* = 0.652; Ohioan, *F*_6,29_ = 2.23, *P* = 0.069), but the values were substantially higher (*F*_1,64_ = 29.35, *P*<0.0005) in the Alaskan frogs (Table 1). The percentage of enzyme present as PKAc decreased during the experiment in both populations (Alaskan, *F*_6,35_ = 4.51, *P* = 0.002; Ohioan, *F*_6,29_ = 13.30, *P*<0.0005), with Ohioan frogs showing an earlier change; however, the percentage of enzyme present as PKAc returned to basal levels during thawing in both populations (Table 1).

**Table 1:** Percentage of enzyme present as PKAc and total PKA activity during freezing and thawing in *R. sylvatica*.

		Alaskan		Ohioan	
Treatment		Percentage of PKAc	Total PKA activity	Percentage of PKAc	Total PKA activity
Unfrozen		25.5±3.1	1.03±0.14	28.8±1.4	0.87±0.05
Frozen	2 h	24.9±4.2	1.08±0.14	39.1±5.4^†^	0.67±0.09
	6 h	31.1±4.7	1.06±0.13	31.2±3.1	0.74±0.12
	30 h	17.7±2.7	1.02±0.11	6.4±1.6*	0.48±0.06^†^
	48 h	15.6±1.7	0.80±0.10	12.4±2.4*	0.65±0.09
Thawed	6 h	10.3±2.1*	1.03±0.13	11.2±3.4*	0.62±0.09
	120 h	22.0±3.9	1.08±0.17	32.1±6.6	0.66±0.05

Values are mean ± SEM (*N* = 4–8). Total activity of PKA is in U mg^−1^ protein. Asterisk indicates that the value differs from the mean for the corresponding sample of unfrozen frogs (Dunnett’s, *P*<0.05); dagger indicates that the value differs between populations (Bonferroni, *P*<0.05).

Total activity of PKA in enzyme preparations incubated at 0°C did not differ (*t* = 1.94, d.f. = 11, *P* = 0.095) between populations, and the values, 0.36±0.05 U mg^−1^ for Alaskan frogs and 0.25±0.02 U mg^−1^ for Ohioan frogs, were ca. 66% lower than those determined for preparations incubated at 22°C. The resultant Q_10_ values for Alaskan and Ohioan frogs, 1.6±0.09 and 1.8±0.10, respectively, were not statistically distinguishable (*t* = 1.87, d.f. = 11, *P* = 0.089).

**Figure 4:**
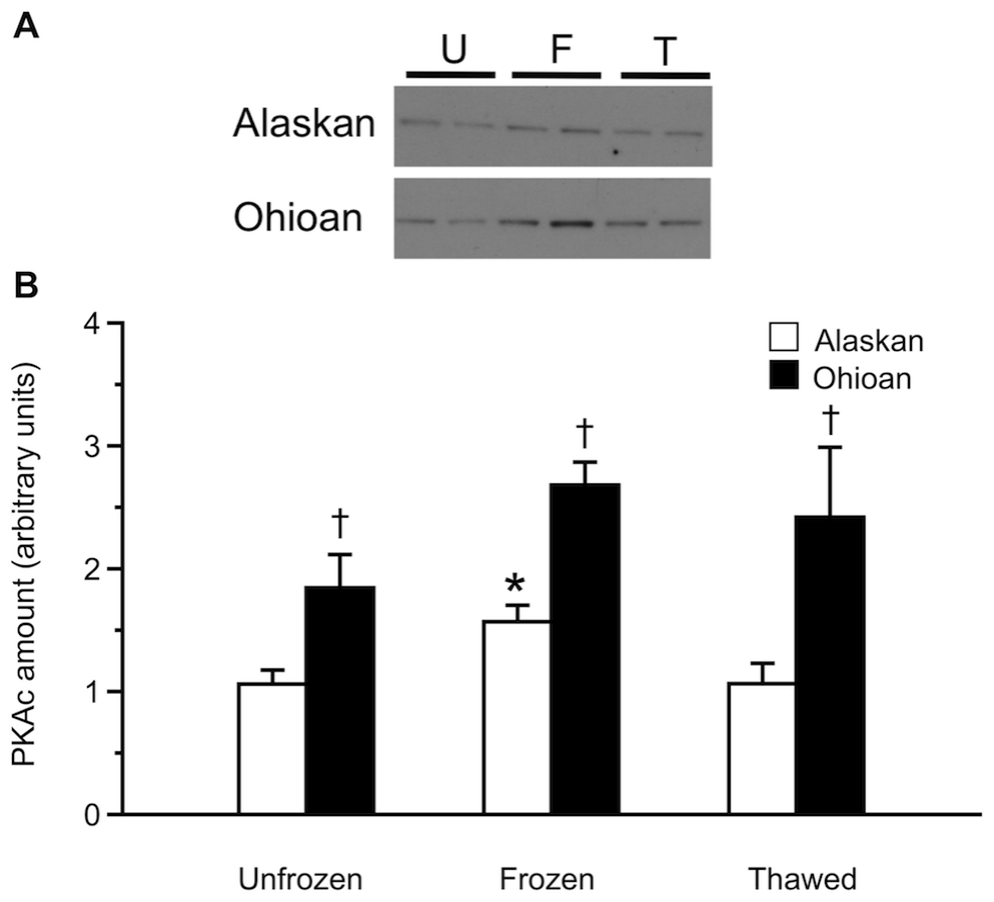
Liver PKAc protein levels during freezing and thawing. (A) Representative immunoblots of hepatic PKAc protein in Alaskan and Ohioan unfrozen (U), 48 h-frozen (F), and 120 h-thawed (T) frogs (2 samples per treatment). (B) Hepatic PKAc protein amounts (mean ± SEM; *N* = 4–8) during freezing (48 h) and thawing (120 h) in Alaskan and Ohioan *R. sylvatica* as compared to that in unfrozen frogs (U). Asterisk indicates that the value differs from the mean for the corresponding sample of unfrozen frogs (Dunnett’s, *P*<0.05); dagger indicates that the value differs between populations (Bonferroni, *P*<0.05).

Incubation with primary antibody for PKAc yielded in all samples a band of ca. 42 kDa, and some samples showed an additional band of ca. 60 kDa. In the competition assay, in which the antibody was pre-incubated with its antigenic peptide prior to incubation with the samples, the 42 kDa band disappeared, whereas the 60 kDa band retained equal intensity; thus, we analyzed only the former, which was specific to the antibody. Immunoblotting results showed that the quantity of PKAc in the liver of Alaskan frogs increased (*P* = 0.025) 1.5-fold with freezing but returned to basal values by 120 h of thawing (Figure 4). In contrast, Ohioan frogs showed no change (*F*_2,13_ = 1.85, *P* = 0.196) in PKAc protein levels. However, these frogs had approximately twice the amount of PKAc protein as did Alaskan frogs (*F*_1,29_ = 29.77, *P*<0.0005).

### Liver GP

**Figure 5:**
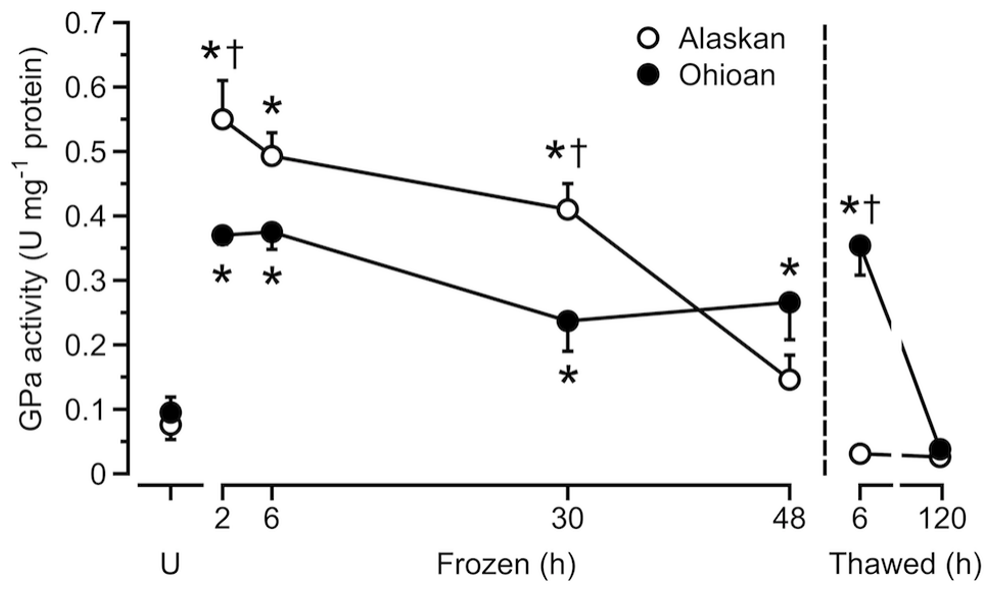
Activity of liver GPa during freezing and thawing. Activity of hepatic GPa (mean ± SEM; *N* = 4–8) during freezing and thawing in Alaskan and Ohioan *R. sylvatica* as compared to that in unfrozen frogs (U). Asterisk indicates that the value differs from the mean for the corresponding sample of unfrozen frogs (Dunnett’s, *P*<0.05); dagger indicates that the value differs between populations (Bonferroni, *P*<0.05).

In the Alaskan frogs, activity of GPa rose with freezing (*F*_6,34_ = 46.87, *P*<0.0005), increasing 7-fold over values for unfrozen frogs within 2 h, but subsequently fell such that it was no longer distinguishable from basal levels by 48 h of freezing (Figure 5). Ohioan frogs also showed an elevated GPa activity with freezing (*F*_6,29_ = 13.35, *P*<0.0005), displaying a 4-fold increase over basal values within 2 h; however, this increased activity was maintained throughout the freezing exposure and even during the early hours of thawing (Figure 5). A two-way ANOVA confirmed this difference in the GPa response between populations, showing a significant interaction (*F*_6,63_ = 13.36, *P*<0.0005) between population and sample treatment. Specifically, GPa activity was similar (*P*>0.99) in unfrozen frogs from both populations, but by 30 h of freezing was about two-fold higher (*P* = 0.007) in Alaskan frogs than in Ohioan frogs. This trend was reversed later in the freezing exposure and by 6 h of thawing Ohioan frogs had the higher (*P*<0.0005) activity (Figure 5).

Overall, changes in both the percentage of enzyme present as GPa and total GP activity reiterated the GPa response observed in both populations. Alaskan frogs showed an increase in both the percentage of GPa (*F*_6,34_ = 58.08, *P*<0.0005) and total GP activity (*F*_6,34_ = 23.93, *P*<0.0005) during early freezing, but by 6 h of thawing, values for Alaskan frogs had returned to basal levels. In contrast, Ohioan frogs showed increases in both the percentage of GPa (*F*_6,29_ = 20.12, *P*<0.0005) and total GP activity (*F*_6,29_ = 6.90, *P*<0.0005), and these only returned to basal values by 120 h of thawing (Table 2). Thus, the pattern of change in the percentage of GPa and total GP activity with freezing and thawing differed between populations (*F*_6,63_ = 15.19, *P*<0.0005; *F*_6,63_ = 7.42, *P*<0.0005, respectively).

**Table 2:** Percentage of enzyme present as GPa and total GP activity during freezing and thawing in *R. sylvatica*.

		Alaskan		Ohioan	
Treatment		Percentage of GPa	Total GP activity	Percentage of GPa	Total GP activity
Unfrozen		30.0±4.2	0.22±0.04	36.0±3.9	0.24±0.05
Frozen	2 h	91.4±1.4*	0.61±0.08*	84.6±2.2*	0.44±0.02*
	6 h	89.4±1.6*	0.55±0.04*	84.2±1.9*	0.44±0.03*
	30 h	81.2±3.8*	0.50±0.04*	68.1±6.9*	0.34±0.05^†^
	48 h	44.5±5.5*	0.30±0.04	71.2±10.2*^†^	0.35±0.04
Thawed	6 h	21.4±3.3	0.14±0.02	82.4±1.2*^†^	0.43±0.06*^†^
	120 h	23.6±5.1	0.10±0.02	26.7±4.8	0.14±0.03

Values are mean ± SEM (*N* = 4–8). Total GP activity is in U mg^−1^ protein. Asterisk indicates that the value differs from the mean for the corresponding sample of unfrozen frogs (Dunnett’s, *P*<0.05); dagger indicates that the value differs between populations (Bonferroni, *P*<0.05).

Total GP activity in enzyme preparations incubated at 0°C was not different (*t* = 0.29, d.f. = 12, *P* = 0.78) between populations. These activity values, 0.017±0.004 and 0.019±0.004 U mg^−1^ for Alaskan and Ohioan frogs, respectively, were ca. 90% lower than those determined for preparations incubated at 22°C. The resultant values for Q_10_, 3.2±0.24 for Alaskan frogs and 3.3±0.14 for Ohioan frogs, respectively, were not statistically distinguishable (*t* = 0.24, d.f. = 12, *P* = 0.82).

**Figure 6:**
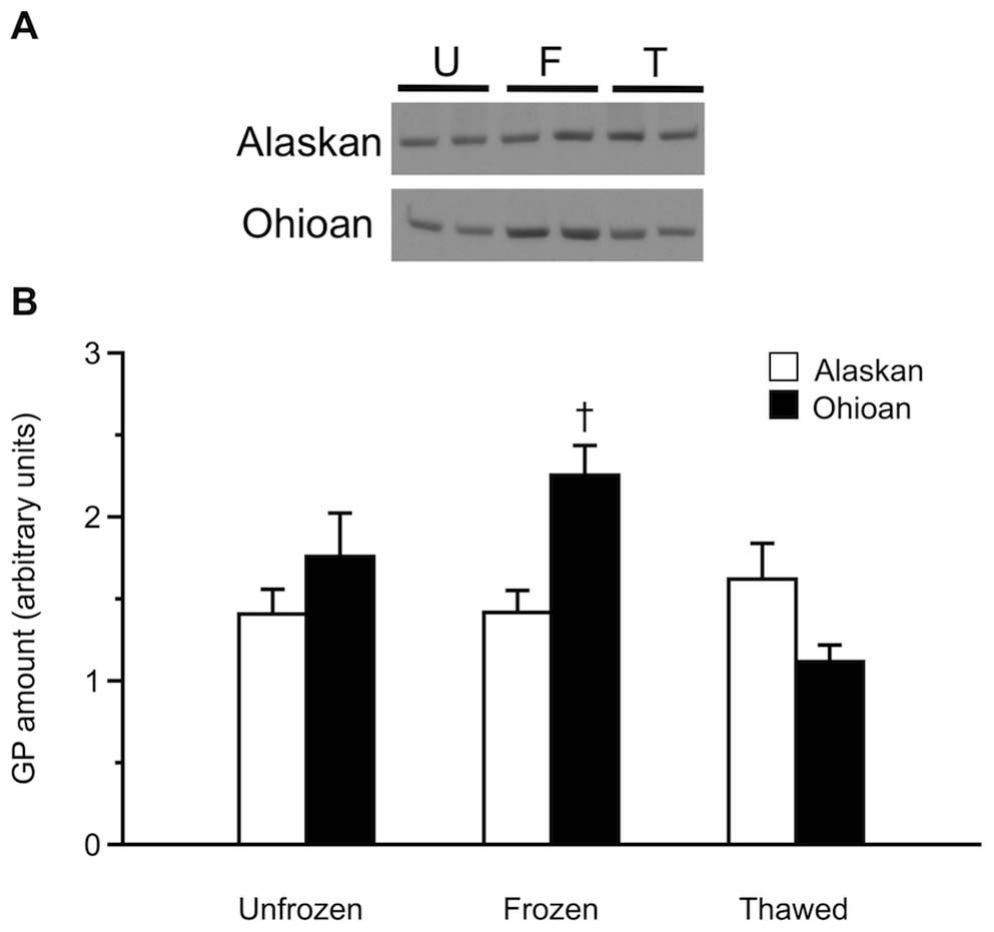
Liver GP protein levels during freezing and thawing. (A) Representative immunoblots of hepatic GP protein in Alaskan and Ohioan unfrozen (U), 48 h-frozen (F) and 120 h-thawed (T) frogs (2 samples per treatment). (B) Hepatic GP protein amounts (mean ± SEM; *N* = 4–8) during freezing (48 h) and thawing (120 h) in Alaskan and Ohioan *R. sylvatica* as compared to that in unfrozen frogs (U). Dagger indicates that the value differs between populations (Bonferroni, *P*<0.05).

Incubation of enzyme preparations with GP primary antibody resulted in a single band of ca. 95 kDa in all samples. In the competition assay, no bands were visible when samples were incubated with the antibody and antigenic peptide, confirming the specificity of the band obtained under standard conditions. In Alaskan frogs, GP protein levels did not vary (*F*_2,16_ = 0.47, *P* = 0.636) throughout the experiment. However, in Ohioan frogs, GP levels did vary (*F*_2,13_ = 5.00, *P* = 0.025) with freezing and thawing, although none of the sample groups differed from values for unfrozen frogs. GP levels in frogs sampled at 48 h of freezing were 1.5-fold higher in Ohioan frogs as compared to Alaskan frogs (*P* = 0.049; Figure 6).

## Discussion

### Glycogen Depletion and Glucose Mobilization

Liver glycogen is the main substrate used by freeze-tolerant frogs when mobilizing the cryoprotectant, glucose, during freezing [1], [4]. We found that Alaskan *R. sylvatica* catabolized their liver glycogen faster than Ohioan frogs during the early hours of freezing, and this resulted in a presumably higher output of glucose for the northern phenotype during this critical period. Alaskan frogs are exposed to lower winter temperatures in their hibernacula as compared to their southern counterparts, and their smaller body size [2] confers them with reduced thermal capacitance, making them especially vulnerable to rapid-freezing injury [16]. Because cryoprotectant distribution becomes severely impaired when higher ice contents are reached [10], quickly mobilizing large amounts of cryoprotectant in the early hours of freezing is likely essential for the survival of this phenotype under subarctic conditions.

We found that Alaskan frogs had larger amounts of glycogen in their livers as compared to Ohioan frogs. It has been proposed that larger hepatic glycogen stores are associated with faster glucose mobilization during freezing [21]. However, in winter-acclimatized *R. sylvatica*, rates of glycogenolysis in hepatocytes *in vitro* are not usually correlated with glycogen content [22]. This has also been observed in chorus frogs [23], but contrasts with the case in other vertebrates, which have more modest glycogen reserves [24], [25]. Nevertheless, as freezing progresses and glucose mobilization proceeds, reduced substrate availability may eventually constrain glycogenolysis rates. The comparatively high glycogen content in Alaskan frogs may obviate or at least defer this constraint, possibly allowing high rates of glycogenolysis to continue for longer periods and, thus, more glucose to be mobilized. The potential for Alaskan frogs to produce even more glucose than they did is evidenced by the fact that they retained a substantial reserve of glycogen (37% of unfrozen frog values) after 48 h of freezing; in contrast, Ohioan frogs retained only 10%. Distribution of the cryoprotectant to non-hepatic tissues is facilitated by their comparatively low ice content and persistence of tissue perfusion during freezing [3].

The exceptionally large store of liver glycogen in Alaskan frogs may also be important in fueling metabolism during the lengthy subarctic winter, which lasts about 9 months of the year [26]–[28], during which time frogs depend on stored nutrients. Presumably freezing episodes in Interior of Alaska are longer than those encountered by wood frogs from more temperate locales [29]. In frozen tissues, cells rely on anaerobic metabolism, which can only be fueled by glucose. The northern phenotype’s larger hepatic glycogen reserve potentially could provide the abundant glucose needed to fuel metabolism during extended freezing episodes. Furthermore, the higher muscle glycogen concentration present in Alaskan frogs [3] may also serve as a source of fuel for anaerobic metabolism during prolonged freezing.

### PKA Response to Freezing and Thawing

Stimulation of β-adrenergic receptors in the hepatocyte membrane triggers a cAMP-mediated activation of PKA within minutes of ice formation, increasing the activity of this enzyme through a rise in the percentage of enzyme present as the free catalytic subunit (PKAc) [4]. PKAc phosphorylates PhK, and this enzyme, in turn, phosphorylates GP, enhancing glycogenolysis [4]. Alaskan frogs potentially could have attained higher glycogenolysis rates by achieving a comparatively large activation of PKAc during the early hours of freezing and/or by maintaining higher total PKA activity. Whereas the latter condition was true, contrary to an earlier finding [7], we did not observe a significant increase in PKAc activity with freezing in either the northern or southern phenotype. In Holden and Storey [7], activity of PKAc rose during the first hour of freezing and dropped afterwards, suggesting that the window to detect the increase in the activity of PKAc is narrow. As our first sample of frozen frogs was not collected until 2 h post-nucleation, it is possible that we missed an earlier, transient increase in PKAc activity. Nevertheless, this enzyme was activated even before freezing began; indeed, the percentage of enzyme present as PKAc (∼ 25%) in our unfrozen frogs was substantially higher than that (∼7%) determined in the earlier study [7]. This disparity may be attributed to the different acclimatization states of the animals: our frogs were winter acclimatized, whereas the frogs used by Holden and Storey [7] were collected following spring emergence. Although PKAc activity was not distinct between the phenotypes during early freezing, the fact that Alaskan frogs maintained a higher total PKA activity throughout the experiment suggests that they have the potential to achieve a superior PKAc activity, and thus potentiate the glucose mobilization response, which perhaps would be evident under more challenging conditions than those used in our study.

As freezing progresses and glycogenolysis proceeds, it becomes unnecessary to sustain high PKAc activity [4]. Accordingly, we observed a decrease in PKAc activity during late freezing in both populations, similar to that reported for Canadian *R. sylvatica* [7]. This change may result from decreased oxygen availability in the tissue due to the ultimate cessation of pulmonary ventilation, freezing of extracellular fluids, and reduced circulation that accompanies progressive freezing [4]. Freezing and anoxia are strong modifiers of PKAc activity, as both independently cause a decrease in PKAc activity in the freeze-tolerant insect *Eurosta solidaginis* [30] and the marine periwinkle (*Littorina littorea*) [31]. Anoxia also decreases PKAc activity in freeze-intolerant species, such as the freshwater crayfish (*Orconectes virilis*) and an anoxia-tolerant turtle (*Trachemys scripta elegans*) [32], [33]. Thawing and the resultant reestablishment of normoxia in various freeze-tolerant animals cause activity of this enzyme to increase to or above control values [7], [31], as was observed in this study. Accordingly, our finding that the decrease in PKAc activity late in freezing was more pronounced in Ohioan frogs supports our conjecture that hypoxia drives the reduction in this enzyme’s activity. Our dissections revealed that Ohioan frogs accumulated more ice during freezing, which presumably would cause their tissues to become more hypoxic, as compared to Alaskan frogs. Indeed, after 48 h of freezing, the plasma of these frogs had 1.5-fold the concentration of lactate as that in Alaskan frogs (22.9±3.3 versus 14.8±1.0 mmol l^–1^, respectively; data from [3]), which further suggests they were more hypoxic. On the other hand, experimental anoxia without freezing reportedly does not influence PKAc activity in *R. sylvatica* [34], suggesting that additional signals may contribute to the regulation of this important enzyme.

Livers of Alaskan frogs had a comparatively higher total PKA activity (Table 1) and yet only about half the amount of PKAc protein as that in Ohioan frogs (Figure 4), implying that the enzyme in the northern phenotype has superior catalytic efficiency. Conducting kinetic assays of purified native enzymes is needed to confirm this contention; however, it is plausible that the putative difference in enzyme efficiency between low and high latitude populations of *R. sylvatica* derives from variation in the PKAc gene or a post-translational modification of the protein [8]. Evolving a more efficient PKAc enzyme could have allowed *R. sylvatica* to colonize Interior Alaska, where subnivian habitats are particularly cold and the demand for rapid, copious production of cryoprotectant is especially great [3]. The occurrence of a regulatory enzyme with distinct catalytic efficiency in different *R. sylvatica* populations would be exemplary among known cases of intraspecific enzymatic adaptation along a latitudinal gradient [35]–[37].

### GP Response to Freezing and Thawing

One common physiological response to freezing in many freeze-tolerant animals is the almost instantaneous activation of liver GP, with a large amount of the enzyme shifting from the inactive form, GPb, to the active form, GPa, through phosphorylation by PhK [38]. This response is not universal, as, for example, in winter, the chorus frog (*Pseudacris triseriata*) maintains a constituitively high activity of GPa (ca. 21 U g^−1^ fresh liver) that is not further increased upon freezing [17]. In *R. sylvatica*, however, basal activity of this enzyme is much lower (ca. 1 U g^−1^ fresh liver), but increases substantially, to ca. 15 U g^−1^ fresh liver, upon freezing [39]. These values, which were determined for *R. sylvatica* from southern Canada, are on the same order as those we recorded for frogs from both Alaskan and Ohioan populations.

Freezing induced a large increase over unfrozen frogs in the activity of GPa (and total GP activity) in both Alaskan and Ohioan frogs. However, in early freezing the activity of this enzyme was significantly higher in Alaskan frogs, as compared to the southern phenotype, for specimens frozen for 2 h or 30 h (Figure 5). Therefore, the faster glycogenolysis seen in Alaskan frogs earlier in freezing was likely driven by the higher activity of GPa measured during the same period. In some freeze-tolerant species, GP activity does not always correlate with rate of glycogen turnover [21], [40], although the two variables are necessarily related. Whereas the increase in GPa activity can be explained by the shift of GPb to GPa [38], the increase in total GP activity was not mirrored by an increase in GP protein, suggesting that post-translational modifications of the protein may have a role in the rise of total enzyme activity [9], [41]. The substantial increase in GPa activity triggered with freezing and the resulting ability to swiftly mobilize large amounts of glucose is likely an essential component of the enhanced freeze tolerance in Alaskan frogs.

In Alaskan frogs, GPa activity (and total GP activity) returned to basal levels by 48 h of freezing, whereas, in Ohioan frogs it remained elevated through the early hours of thawing. Preservation of the hyperglycemic state during freezing is achieved through a bypass of the usual glucoregulatory mechanisms and maintenance of elevated GP activity in the liver [4]; thus, the drop in GP activity in Alaskan frogs later in freezing suggests that such mechanisms were somehow lost. Possibly, this change reflects the action of insulin, which lowers glycemia through both direct and indirect action in the liver [42]. Wood frogs from more temperate populations exhibit an increase in serum insulin level during freezing [43], but liver glycogenolysis nevertheless continues and GPa activity remains elevated even after thawing [44]. Conceivably, suspension of glucoregulatory control could stem from inhibition of the hormone’s action, as is seen in mammalian tissues when intracellular dehydration induces insulin resistance [45]. However, the specific mechanism by which such inhibition is putatively released late in the freezing of Alaskan frogs is unknown. It would be instructive to determine if these frogs, when frozen to temperatures lower than those used in our study, would longer maintain elevated GPa activity and thereby more fully convert their glycogen reserve to the cryoprotectant glucose.

### Effect of Temperature on Enzyme Assays

Because the cryoprotective glycemic response is triggered by the onset of freezing [1], [4], the physiologically relevant temperatures for the enzymatic processes under investigation lie within a narrow range extending just below the equilibrium freezing point of body fluids, ca. −0.6°C. However, given the convention of investigating kinetic phenomena at room temperature, and in order to compare our data with published values, we performed most of our assays at 22°C. We nevertheless conducted some tests at 0°C to determine if using the higher temperature confounded comparisons between the phenotypes, which could occur if, for example, the native enzymes had different thermal optima. Our findings showed that neither the total PKA activity nor total GP activity at 0°C differed between phenotypes; thus, we are reasonably confident in the conclusions drawn from the results of assays conducted at 22°C.

Comparing Q_10_ values for total PKA activity and total GP activity showed that the thermal sensitivity of both enzymes was similar between populations. The Q_10_ values for total PKA activity in liver homogenates from Alaskan and Ohioan frogs were 1.6 and 1.8, respectively, similar to that determined for PKA purified from *R. sylvatica* [46]. Thermal sensitivity of total GP activity is comparatively greater, as the Q_10_ for this enzyme, known from other ectotherms, ranges from 2.5 to 10 [47]–[49]. Accordingly, Q_10_ values for total GP activity from our Alaskan and Ohioan frogs were 3.2 and 3.3, respectively. A study of hepatic glycogenolysis rate as a function of temperature showed the Q_10_ for the process was 1.98 [50], a value similar to our values for total PKA activity but lower than the Q_10_ for total GP activity. Thus, there must be cellular mechanisms that compensate for the relatively high thermal sensitivity of GP, since the overall glycogenolytic pathway seems to be less affected by temperature than this enzyme, which is essential to glycogenolysis.

### Protein Responses

The ability to change the expression levels of certain enzymes and other proteins is an essential response conferring local adaptation and stress tolerance [51]. In devising our immunoblotting protocol, we determined that, although freezing had no effect on β-tubulin levels in liver, Alaskan frogs contained twice the amount of this protein as did Ohioan frogs. Microtubules, polymers of α- and β-tubulin, are the major component of the cytoskeleton, and, in addition to serving a structural role, function in regulating cell volume and cold sensing [52]–[54]. The greater abundance of β-tubulin in Alaskan frogs may contribute to their ability to survive freezing to extremely low temperatures, although more research is needed to probe the possible contribution of this protein to freezing survival in this and other freeze-tolerant species.

Changes in gene and protein expression are common responses in freezing adaptation in myriad organisms. In *R. sylvatica*, freezing-responsive genes include ones involved in metabolic suppression, cell repair, and antioxidation, as well as those encoding novel proteins [55]. Despite the importance of the glycemic response to freezing survival, no study to date has documented freezing regulation of the enzymes involved in glycogenolysis. Indeed, levels of the GP protein in livers of both Alaskan and Ohioan frogs remained unchanged with freezing, which is consistent with results for frogs of a Canadian population [9]. We did, however, find an increase in the abundance of PKAc that was significant for the Alaskan population. Such a response in the early hours of freezing may enhance the freezing mobilization of glucose by potentiating GP activation. Curiously, the greater abundance of PKAc was not associated with an increase in total PKA activity; however a mismatch between protein levels and enzyme activity is potentially explained by mechanisms such as inhibition of enzyme activity by the natural inhibitor, PKI, or glutathionylation of specific residues [56], [57]. PKAc is involved in the transcriptional regulation of several genes, including those involved in metabolism and cell-cycle regulation [58]. Consequently, the increased abundance of PKAc during freezing may also reflect a shift in the transcriptome of hepatocytes.

### Conclusion

In this study, we determined whether the enhanced freeze tolerance of Alaskan *R. sylvatica*, which can survive freezing at temperatures at least as low as –16°C [3], potentially could derive from distinct freezing responses of hepatic enzymes, key to the glucosic cryoprotectant response. Relative to Ohioan frogs, which tolerate freezing only to ca. –5°C [5], Alaskan frogs catabolized liver glycogen faster during the early hours of freezing by maintaining a higher activity of GPa, and maintained higher total PKA activity throughout the freezing time course. Furthermore, Alaskan frogs modulated their glycogenolytic response by decreasing the activity of GPa to basal levels after freezing was underway, which presumably allows them to retain a substantial reserve of glycogen during moderate freezing episodes. This contrasted with the response of Ohioan frogs, which maintained a high activity of GPa throughout freezing and consumed nearly all their liver glycogen. The unique hepatic responses of Alaskan *R. sylvatica* likely play an important role in this phenotype’s exceptional freeze tolerance, which is essential for the survival of this species in regions where winter conditions are so extreme.
